# Loneliness Among Older Adults Receiving Home Care: A Phenomenological-Hermeneutical Study

**DOI:** 10.3390/nursrep16070230

**Published:** 2026-07-02

**Authors:** Birgit Hauger, Randi Martinsen, Knut Hestad, Liv Skomakerstuen Ødbehr

**Affiliations:** 1 Department of Health and Nursing Sciences, Faculty of Social and Health Sciences, University of Inland Norway, 2406 Elverum, Norway; randi.martinsen@inn.no (R.M.); knut.hestad@inn.no (K.H.); liv.odbehr@inn.no (L.S.Ø.); 2 Department of Research and Innovation, Innlandet Hospital Trust, P.O. Box 104, 2381 Brumunddal, Norway

**Keywords:** home care, home dwelling, loneliness, nursing, older patients, phenomenological-hermeneutical, qualitative

## Abstract

**Background/Objectives:** Norway’s ageing population includes many older adults living alone who receive home care and are at increased risk of loneliness. Loneliness is the subjective sense of unmet or imbalanced social needs, shaped by culture and living conditions, and can be social (lack of contact) or emotional (absence of close, trusting relationships). In older people, it often follows partner or role loss or reduced mobility or participation and is associated with emotional pain, lowered self-worth and poorer health and quality of life. This study aimed to explore patients’ experiences of loneliness while living alone and receiving home care. **Methods:** Twelve older patients (aged 74–98 years) participated in in-depth interviews, which were analysed using phenomenological-hermeneutical analysis in line with Lindseth and Norberg’s recommendations. **Results:** The results are presented under the following themes: (I) An overwhelming and painful feeling, (II) A presence without connection, and (III) Experiencing a sense of alienation. **Conclusions:** This study describes complex feelings of loneliness for the majority of participants, often worsened by poor mobility and shrinking social networks. From the patient perspective, good home care goes beyond practical and medical tasks: patients need to be treated as whole persons, with respect and understanding, to alleviate loneliness. Staffing stability, predictable visiting times, time for conversation, and small acts of kindness are central to well-being and the prevention of loneliness. Municipal healthcare should prioritize relationship-building, communication skills, and organizational solutions that enable continuity and flexibility. Focusing on the patient perspective in planning and evaluation will create better targeted interventions and support dignified ageing.

## 1. Introduction

Worldwide, life expectancy is increasing [1], and it has risen significantly in recent years [2]. This development is expected to continue in the coming years [3]. Norway, like many other countries, is facing an ageing population, with a growing number of older adults living alone [4]. Living alone refers to residing without others in the household and is often linked to higher risks of physical and mental health problems, as well as social isolation and mortality [5]. Living alone has been associated with a 21% higher risk of all-cause mortality among older adults [3]. Importantly, however, living alone does not necessarily cause negative health outcomes, and any adverse health effects of living alone can be reduced by factors such as social support and neighbourhood cohesion [5]. Older adults encounter both positive and negative aspects of solitude. Living alone and spending time alone are typically experienced more positively when they are voluntary and characterized by autonomy [6]. Many older adults intentionally choose solitary living to preserve their independence and self-identity, seeing their homes as spaces that support their identity and give them a sense of purpose [7].

Loneliness can be described as a subjective experience in which a person’s social needs are not satisfied or are out of balance [8]. At the same time, loneliness is shaped by cultural expectations and daily living; what counts as “closeness” or an “adequate social network” varies across cultures. In some societies, feelings of loneliness are tied to reduced mobility or an inability to travel or work, which can create a sense of uselessness [9].

Social loneliness is described as a lack of contact with others, while emotional loneliness concerns the absence of close, trusting relationships [8]. Older people often describe loneliness as being related to the loss of a life partner, friends or a social role, as well as limitations in mobility and participation in activities. These experiences are often accompanied by emotional pain, low self-esteem and a reduced sense of self-worth [10]. This can negatively affect health and quality of life [11]. These forms of loneliness overlap and are interrelated. A person can have many social contacts and still experience emotional loneliness, or one can lose social participation without feeling an emotional lack. 

Existential loneliness is described as a third subtype of loneliness and is particularly relevant for older people because it is connected to the awareness of life’s end. Older people who themselves are approaching the end of life or who have peers in that situation may be particularly vulnerable to experiencing existential loneliness [12]. Existential loneliness can be described as distance from people and distance from the world. This experience can trigger intense negative feelings such as isolation, alienation, a sense of emptiness and the feeling of being abandoned. Awareness of one’s separateness can also occur without intense negative feelings or may even be experienced as insightful or constructive. The core of existential loneliness lies in the encounter with one’s own separateness [13].

Loneliness can arise from underlying predispositions such as introverted personality traits, a history of trauma, or chronic mental health conditions, and may also be triggered by specific life events, such as loss of mobility or health, retirement, or the end of important relationships [8].

The prevalence of loneliness is highest among those aged 80 and above [14], and loneliness negatively affects quality of life in old age [15]. Older people who feel lonely are more likely to report poor health, reduced physical functioning and various chronic conditions [16,17]. Loneliness and reduced social interaction are associated with various adverse health outcomes, including symptoms of depression, a heightened risk of dementia, and an increased likelihood of premature death [18]. However, the burden of loneliness is experienced differently [19]. A cohort study found that higher loneliness at baseline was associated with greater depressive symptom severity over up to 12 years of follow-up. The relationship between loneliness and depression may be bidirectional: loneliness can increase the risk of depression, and depressive symptoms can increase the risk of loneliness [20]. Another cohort study found that the subjective feeling of loneliness was associated with an increased risk of dementia after three years, whereas objective indicators of being alone did not show an independent effect after adjustment. This finding suggests that perceived loneliness may be an important early signal or risk factor for dementia. Loneliness could reflect pre-clinical dementia due to reduced cognitive stimulation and, thus, lower neural reserve, or it could represent other risk factors. This indicates that loneliness may have long-term consequences for brain ageing and cognitive health [21]. Many older adults living alone receive home care to assist them in daily life. With the number of older adults significantly increasing [2], the trend of a substantially higher proportion of older individuals who require home care will increase in the coming years. Home care in Norway encompasses a broad range of health-related services delivered by healthcare assistants and registered nurses in the patient’s home and is mandated by Norwegian legislation [22]. Home care traditionally provides medical treatment, nursing and daily living support in the patient’s home to promote safety, independence, quality of life and continuity of care, including follow-up after hospital stays, management of chronic conditions, disease prevention, and support for physical and mental well-being. In principle, it represents a flexible and individualized approach, aiming to enable patients to remain at home in a safe and appropriate manner [23].

The aim of this study was to explore patients’ experiences of loneliness while living alone and receiving home care.

## 2. Methods

### 2.1. Study Design

This study has a qualitative design with a phenomenological-hermeneutical approach [24] to explore older patients’ experiences of loneliness while living alone and receiving home care. Individual in-depth interviews with older patients from eight municipalities in Norway were conducted to gain deeper insight into how they experienced living alone when receiving home care services.

### 2.2. Setting and Recruitment of Participants

To be included in the study, participants had to be patients over 65 years of age, living alone at home, receiving home care services, and able to give informed consent. A further inclusion criterion was that participants could express themselves verbally and provide detailed descriptions of their experiences. Participants who received home care but lived with a spouse or other family members did not meet the study’s inclusion criteria. Invitations to participate were sent to municipal leaders and home care managers in seventeen municipalities, together with an explanation of the study aim, and a request for their consent to conduct the study and for their assistance in recruiting patients. Eight of the municipalities responded to the request and wished to participate in the study. The researcher (BH) provided clear and explicit instructions about the study’s inclusion and exclusion criteria. After agreeing on the recruitment procedure, the home care managers in each municipality took responsibility for selecting participants for the study. The home care managers were asked to give written information about the study to suitable candidates for participation in accordance with the inclusion criteria. A total of 19 patients receiving home care received oral information about the study, and those who wished to participate were given additional written information (12 participants). The home care leaders informed the researcher about the patients interested in participation, provided these patients with the consent forms, collected the signed forms, and subsequently handed them to the researcher. Following this, the first author contacted each participant individually to arrange a suitable time and place for the interview. All participants were asked where they preferred the interview to take place, and all chose to have their interview conducted in their home. Five patients declined to participate without giving any reasons. Two participants initially agreed to take part and signed the informed consent form, but when contacted by the researcher to schedule an interview, they decided against participation. The reasons were that one of them had changed his mind, while the other said that she was too frightened to take part in the interview.

### 2.3. Descriptions of the Participants

The participants received home care due to various physical conditions and therefore needed help to manage their health and continue living alone at home. Some reasons for receiving home care were help with administering eye drops, dispensing medications, putting on compression stockings, nutrition, or morning care, such as personal hygiene and getting dressed. The frequency of home care visits varied from one to four times a day. None of the participants received home care due to mental health reasons, and none had been screened for cognitive impairment because this was not relevant. Eight participants used a walker and several of these had severe difficulty in walking.

Finally, a total of twelve patients from eight municipalities were recruited for the study. Seven female and five male patients from eastern Norway participated; their ages varied from 74 to 98 years. They had received home care for periods ranging between one and 39 years, with a mean duration of 7.9 years. Ten of the participants had previously lived with a spouse/partner but had now been living alone for periods ranging from three to 35 years. Two participants had lived alone their entire adult lives. Seven participants were urban dwellers, while five were living in rural areas. Table 1 gives an overview of some characteristics of the participants.

### 2.4. Data Collection

The twelve individual in-depth interviews were conducted from 13 March to 26 March 2025. A thematic guide supported the interviews, including topics relevant to the participants’ lived experience of the phenomenon of loneliness [24]. The thematic guide included the following main statement: “Please tell me about your experiences of loneliness while living alone and receiving home care”. The interviews followed a methodological approach that allowed the participants to speak openly about their experiences of loneliness. For example, participants were asked follow-up questions, such as, “Could you elaborate on what you just mentioned?” and “Could you expand on that?” In addition to posing follow-up questions, the researcher was attentive to maintaining open and receptive body language in order to encourage openness and demonstrate interest in the participants’ narratives.

The interviews lasted between 58 and 77 min and were audio-recorded and then transcribed verbatim. The audio files and transcripts were then securely stored in the University of Oslo’s cloud storage solution, Services for Sensitive Data (TSD) [25]. Participants were offered the opportunity to review their transcribed interviews (member checking), and one participant used this option, but without commenting on the interview. Theoretical saturation was judged to have been reached when no new themes emerged from additional interviews, as assessed through team discussions.

### 2.5. Data Analysis

The study was inspired by phenomenological hermeneutics to describe the participants’ experienced lifeworlds and interpret the meaning of these experiences in their context. The lifeworld refers to the immediate, everyday world as it is subjectively experienced by individuals [24,26]. Phenomenology attends to lived experience and how lifeworlds are revealed through experiences [26], while hermeneutical methodology emphasizes understanding and interpretation [27,28]. An advantage of phenomenological-hermeneutical analysis is its dynamic interplay between understanding and explanation [29], with a focus on orientation towards the phenomenon [30]. Phenomenological-hermeneutical analysis combines a phenomenological description of lived experiences with a hermeneutical interpretation of their meaning [29]. Conversation and interaction with participants are important aspects [31].

The transcribed texts were analysed with inspiration from phenomenological-hermeneutical analysis as described by Lindseth and Norberg [24]. An inductive approach was employed, focusing on the phenomenon in the data [32]. The analysis consisted of three steps: (i) naïve reading, (ii) structural analysis, and (iii) comprehensive understanding. Naïve reading refers to approaching the text as a whole and allowing it to speak for itself, with the aim of forming an intuitive, preliminary understanding of the text. All the interviews were read in their entirety several times to gain an overall sense of the text [24]. Analytical transparency was enhanced by keeping detailed notes of coding iterations and team discussions, which helped to clarify how interpretations were developed and refined.

The selected quotations were chosen for their ability to illustrate core themes and variation across participants’ experiences (anonymized). Meaning units were identified as distinct text segments conveying a single idea. These were condensed without losing core meaning and grouped iteratively into sub-themes and themes. Coding was performed by BH, and emergent codes and themes were discussed and revised in the team of supervisors (RM, KH, LSØ) throughout the analysis. The researcher’s preconceptions were documented in reflexive notes after each interview and explicitly addressed in discussions in the team of supervisors, where alternative readings were sought to reduce confirmation bias. Analysis was conducted iteratively alongside data collection. The interpretations are context-dependent and shaped by the researcher’s perspective, aiming to enhance transparency.

There were continuous reflexive processes, discussions of preconceptions, research memos, and critical exchanges of views within the research team. These research meetings were pivotal for developing more nuanced themes that better reflected participants’ perspectives and the complex interplay between the relational factors involved. Disagreements were discussed in the research team. Themes emerged through several rounds of analysis and reflective meetings. The research team asked critical questions, such as “Could this be interpreted differently?”, questions that prompted deeper reflection and iterative movement between the parts and the whole during the analysis.

Structural analysis is a way of interpreting a text by identifying themes that capture the core meaning of the phenomenon under study [29], in this case, the patients’ lived experiences of loneliness while living alone and receiving home care. First, the text was broken down into meaning units [24]. After the meaning units were selected, they were read and compared with the naïve reading, and their content was condensed while preserving the essential meaning. This condensation process involved rewriting the key ideas in more everyday language. It is important to carefully consider the selection of meaning units in relation to the phenomenon being studied [24]. This process involves constant movement back and forth between the whole text and its parts [32]. Next, the condensed meaning units were reviewed to find patterns that could lead to the formation of sub-themes [24]. The sub-themes were grouped into broader themes and then into main themes [32]. The themes represented a description of meaning drawn from the text. During this process, the meaning units were temporarily removed from their original context to be analysed. Finally, the themes were validated against the naïve reading to ensure that they made sense and stayed true to the overall impression of the text [24].

The sub-themes and themes were formulated in relation to the aim and context. This interpretation aimed to stay as close as possible to the actual lived experience, using everyday language. This process, including reflection and discussion among the research team, resulted in the identification of three themes, which are presented in the analysis section [24]. See Table 2 for examples of the analysis process.

A comprehensive understanding and a phenomenological-hermeneutical interpretation of the lived experience were then discussed, where the researchers’ pre-understanding was carefully considered.

### 2.6. Ethical Considerations

The Norwegian Agency for Shared Services in Education and Research (SIKT) [33] granted approval to conduct the study (No. 467880/2023). The study was also approved by the Research Ethics Committee of the University of Inland Norway (No. 24/09643), dated 13 March 2025 [34]. The guidelines for risk and vulnerability analysis of the General Data Protection Regulation were followed [35]. An electronic platform at the University of Oslo was used to store data. This platform enables the collection of sensitive information for the Services for Sensitive Data system (TSD), which meets the strict legal requirements for digital processing and storage of sensitive research data [25]. The study was conducted in accordance with the Declaration of Helsinki in that the in-depth interviews were based on voluntary, written informed consent, anonymity, protection against harm, and the opportunity to review and withdraw from the study. Potential participants received written and oral information before the study and those who wished to take part received and signed an informed consent form before participation. Efforts were made to ensure that participants felt listened to and respected throughout the process [36].

## 3. Results

The results are presented below, describing the phenomenon of older patients’ experiences of loneliness when living alone and receiving home care.

### 3.1. Naïve Reading

Feeling alone when living alone was often felt like a heavy weight that shaped the whole day for the majority of the participants. As many as ten out of twelve participants experienced loneliness as tough and painful, filling hours with emptiness and doubt about the point of continuing to live. Losses of a partner, friends and mobility shrunk their world, leaving few opportunities for meaningful contact and a persistent sense of being unseen rather than known as a person. Being noticed and treated as a “whole person” was a crucial means to ease that loneliness. Small, considerate acts by home care staff such as taking a break to talk or helping with a practical task made the patients feel seen and acknowledged. However, rushed, task-focused visits, unpredictable arrival times and rotating staff reinforced feelings of invisibility and worthlessness for most of the participants. When care felt like a checklist, loneliness and alienation worsened.

Loneliness was also shaped by practical realities: reduced mobility, long waits for scheduled visits, and staff shortages increased the sense of insecurity in most participants. When care was consistent, empathetic and respectful, all the participants described greater reassurance and less distress. Where care felt indifferent or impolite, all participants reported intensified isolation and a diminished sense of dignity.

For two participants, living alone was an active, positive choice that provided freedom, joy of life and autonomy. At the same time, all of them experienced a sense of security from visits by the home care service, and the quality of the visit determined whether it felt reassuring or created distance.

### 3.2. Structural Analysis

The structural analysis of the interviews is presented based on the following themes: (I) An overwhelming and painful feeling, (II) A presence without connection, (III) Experiencing a sense of alienation.

#### 3.2.1. An Overwhelming and Painful Feeling

The results indicate deep despair, doubts about the meaning of life, and feelings of being overwhelmed by loneliness:

“I have just one wish, which is that I could stop struggling with all this pain and just be allowed to die. There’s no point in me being here. No one cares, and I sit here completely alone with nothing to do. It’s not a dignified life, and it makes no sense to me. I feel very lonely and that’s difficult and sad”.

This study identified widespread experiences of loneliness and limited social contact with others. It became clear that, as people age, many lose close friends, and family members often live far away:

“Sometimes it gets so lonely, you know. You become so lonely, and it hurts. It all started when my wife died. I guess I’m a bit depressed, I must be. It’s the loneliness that’s causing it, actually, and I feel a low quality of life. That’s obvious. I miss someone to talk to and be seen for who I am, not just an old patient. My only son lives on the other side of the country, so I don’t see him so often”.

The emotional and psychological burden of loneliness was experienced as a heavy, intrusive state that shapes a person’s entire daily life: “So, the day passes, but the loneliness is overwhelming and painful. That’s how I feel. For me, the loneliness takes up a lot of space. I wish home care would like to get to know the real me and have conversations with me”.

Ten of the twelve participants experienced insufficient meaningful communication and presence from home care staff, which was associated with feelings of loneliness. Care quality varied considerably, and differences in staff communication and treatment, ranging from condescending or harsh interactions to respectful and satisfactory encounters, shaped the participants’ experiences of loneliness.

A few of the patients described the burden of long-standing, heavy doubts about the point of living, but they were unable to share these thoughts with the home care staff:

“I often think, ‘Why have I lived so long? Am I just a burden now? Or why am I living this long?’ I can’t talk about these thoughts with the home care nurses, but I often have these thoughts. ‘Is there any point in getting old?’. I have little quality of life due to my illness. I’m alone and have little contact with others. Not because I don’t want to, but you can’t expect people to constantly come and ask how I’m doing. My quality of life has really gone down. I also feel like I’m not the same person since I started having problems with walking. My mobility is very limited”.

This illustrates the complex interplay of ageing, limited mobility and loneliness. It highlights the internal conflicts faced by individuals who struggle to find meaning in later life while coping with loneliness. Physical functioning and mobility play a key role in the experience of loneliness. However, living alone was also experienced as positive and described as a deliberate choice by two of the participants. It provided a sense of independence, calm and control over daily life. One of the participants said, “Living alone is so wonderful. I can do whatever I want. I make decisions about myself and my time, and I have the freedom to do exactly as I please. I’ll never live with anyone again; I’m done with that. I live alone, but I’m not lonely”.

#### 3.2.2. A Presence Without Connection

This study highlights that respectful, empathetic and attentive care is essential for fostering trust and a sense of security in home care patients. There was considerable variation in participants’ experiences of the empathy and communication displayed by home care staff. In some cases, home care was found to be empathetic and professional, as expressed in this typical example: “They’re outstanding; they call to check on how I’m doing, and they follow up well. They arrive at my home as quickly as they can, often out of breath. They’re truly remarkable, respectful and compassionate. Yes, they are”.

However, while empathy was sometimes present, a lack of meaningful connection with home care was common. Some participants often experienced home care as routine and task-focused rather than truly caring: “They come, administer the medicine, and leave again. There’s really no relationship with the home care staff. They don’t really understand who I am, what I feel, or what I need. I can’t talk to them about how I’m feeling, and I never do either”. Staff would come, but with little time to listen or connect; visits felt rushed and sometimes impolite. As a result, most of the participants felt unseen and misunderstood, unable to talk about their feelings or needs and left without the human contact that would make them feel supported. The participants’ experiences indicated a sense of being treated as a task rather than a whole person, resulting in diminished personal connection and limited genuine interest in who they were:

“Many of them shouldn’t be nurses. They have little empathy for patients. They seem like they don’t care. When they enter the house, they should be a bit polite. They could at least present themselves and explain what they’re going to do. I dread every morning and evening, thinking: ‘Who’s going to come now?’ Because I never know who will turn up. So, I have to accept whoever comes. Maybe this is typical of a big city, with lots of different healthcare staff coming, and a lack of continuity, as it’s never the same people.”

The majority of the participants found that home care staff were often in a rush and tended to perform only those tasks required by their formal duties. Reports of care going above and beyond, doing “a little extra”, such as taking out the rubbish, folding clothes, or sitting down to talk, were associated with feelings of being seen and a reduced sense of loneliness:

“I don’t have any specific requests for the home care staff, because I don’t receive much care anyway. But if there’s something I need assistance with, there are a few of them who would help me, such as taking out the rubbish. But I can’t tell anyone, because they’re not allowed to help me with practical things. Some also respond to me in an unfriendly way, as if they were irritated when I ask them to help me with other things than they’re supposed to help me with. It would mean a lot to me if they’d assist me since my hands no longer function properly.”

Loneliness was worsened by uncertainty about arrival times. Unpredictability and prolonged waiting created an impression of not being prioritized and of being unseen, intensifying feelings of isolation. The study identified some dissatisfaction with home care staff, including a perceived lack of empathy:

“I feel like they’re looking down on me. They enter quickly and just do what is needed. I need eye drops put in, and the home care staff must stay in my home for 15 min to observe the effect. During that time, some of them pick up their cell phones and just sit here, busy with their phones. I wish they could talk to me instead”.

Being spoken to in an unfriendly manner was experienced as being looked down upon, and increased the feeling of loneliness: “Once, the home care staff said to me when I asked why they were two and a half hours late to make me breakfast: ‘Well, we can’t let people die in our arms just so you can have hot coffee.’ Hearing that made me feel so insignificant. There’s such a huge difference between them, honestly.”

#### 3.2.3. Experiencing a Sense of Alienation

The study found that the availability of home care provided a sense of security by offering support when needed. However, it also revealed some differences in the quality and accessibility of services:

“The fact that home care comes gives me a sense of security. If something happens, if I have questions, it feels like security for me. They come and check how things are going. If something suddenly happens, they can call the doctor, for example. I’m very grateful that home care comes. I used to live in the city, where several staff members would come at different times. After moving to the countryside, I now have fewer home care staff to interact with, and I find they’re friendlier here. It feels more secure in the countryside…When I feel safe, I don’t feel so lonely”.

A few of the participants felt they were being judged and talked to in a strict tone. Such experiences contributed to a sense of disconnection and diminished dignity: “Sometimes I feel they are too strict, pushing me to exercise as if I’m not doing it. Once, I was asked to make a sandwich and I felt like I was being tested, as if I didn’t deserve the help. That gave me a bad feeling”.

The study found widespread frustration in many of the participants due to limited time and resources in home care, negatively affecting care quality and the ability to get answers to questions. Home care staffing levels were perceived as insufficient, with temporary staff, often lacking adequate training and experience, frequently delivering care. Loss of control and weakened social connections in daily life were reported, combined with the need to adapt to the schedule of home care. There was a clear desire for greater freedom and flexibility in how time was managed:

“I thought I could stay in bed as long as I wanted when I was this old, but that’s not possible when home care arrives. I feel I must be up and dressed, which makes every day feel the same. It’s like wearing an ankle tag. I always have to be present, and the timing varies from day to day. I’ve spent a lot of time waiting for them. And when they come, they rush out again without even talking to me or asking me how I’m doing. I feel like a task that needs to be done, not like a person anymore”.

The results show that home care staff were frequently perceived as pressed for time by most participants, with recurrent long waits for scheduled visits: “One day, I called around 11 o’clock to ask if I was going to get morning help. They didn’t come until ten past two. That was the first help I got that day”.

The results reveal a strong desire for conversations with home care staff in almost all participants. Staff shortages were linked to lower quality of care and difficulty in getting answers to questions:

“There are too few home care nurses, and many rely on temporary staff with little experience and varying levels of competence. They often take the job for extra income rather than wanting to make a difference and they can’t always answer my questions. I need more consistent care. I don’t think the municipality cares about older people like me, so there are no services like social contact, a daycare centre or time for conversations”.

The majority of the participants raised concerns about the large number of different home care staff visiting, which increased their feelings of loneliness. Frequent rotation of staff made it difficult to establish familiarity, creating distance and heightening loneliness and alienation. Variations in staff competence were also noted, with many home care workers perceived as lacking experience in home care, undermining the participants’ sense of security. Conversely, satisfaction with care, as reported by some of the participants, was associated with reduced experiences of loneliness.

## 4. Discussion

This study provides new, specific insights into how the context of home care shapes patients’ experiences of loneliness. The results show that loneliness was perceived as due not only to a lack of social contact but also to the attitudes of home care staff, where participants felt judged, experienced a lack of connection with staff, or were ignored as human beings. These factors may be expected to strengthen the sense of loneliness. The data indicate loss of autonomy and dignity as central mechanisms in the experience of loneliness. Small everyday events, such as feeling “tested” during routine tasks, can undermine self-worth and make people withdraw or avoid closer relationships. The study also nuances the concept of loneliness among older people living at home by distinguishing between relational disconnection, existential loneliness, and perceived professional distance in care encounters. This differentiation can enable interventions to be tailored to specific aspects of loneliness. There are clear clinical implications for the management and practice of home care. The results highlight the importance of a person-centred approach, allowing enough time during visits, and making small changes that can strengthen patients’ dignity and sense of belonging.

In this study, loneliness emerged as a pervasive physical and emotional experience that highlighted the importance of being seen, heard and acknowledged not only as patients but also as individuals. Further, a desire to be met with understanding and respect was expressed. These are clearly elements that reduce feelings of loneliness. At the same time, living alone was not universally experienced as loneliness. For some, solitude was a deliberate choice that brought independence, calm and freedom. Thus, loneliness and living alone are not synonymous; the difference often came down to connection, continuity and the simple presence of someone who cared. The lack of these factors leads to a form of emotional loneliness characterized by the absence of a close, caring and continuous relationship. In short, meaningful human contact, sustained attention, courtesy and staff continuity emerged as the primary means to be seen, to feel safe, and to feel less alone.

Home care staff were perceived as warm and respectful in certain contexts, while in other settings, staff were reported as rude, lacking empathy, or dismissive. These negative interactions led some patients to dread the arrival of home care and increased their feelings of abandonment and loss of control, reflecting emotional loneliness arising from a lack of empathetic, trusting relationships. Similar results have been reported in other studies, which emphasize that inconsistent communication and unprofessional behaviour can compromise patients’ sense of safety. However, in one of these studies, there was a small sample size [37]. Being acknowledged and met with respect and good communication has a significant effect on patients’ experience of home care and helps to establish a positive relationship with staff [38,39]. A study shows that being met with indifference and being unable to share one’s life fosters existential loneliness among frail older adults, which may explain why more connected, supportive care could alleviate loneliness. However, this particular study has a small, context-specific qualitative design (*n* = 23) and does not constitute empirical proof that specific interventions will reduce loneliness in wider populations [40]. Existential loneliness lies on a deeper level in patients’ lives and touches on their philosophy of life, experience of meaning, faith and thoughts about death. It requires more of home care staff to address existential loneliness due to their probable lack of preparedness to meet such questions and the time frame available in home visits. It is easier to address social and emotional loneliness due to their visibility. Limited mobility often leads to isolation, frequently stemming from poor physical health, particularly difficulty in walking. This type of isolation may increase the feeling of separation from the world and thus heighten existential loneliness [13,40,41,42]. This finding aligns with research showing that good physical health, by enabling movement, getting outdoors and socializing, is a key determinant of well-being and provides a sense of freedom and autonomy [43,44]. Several participants in this study described deep feelings of hopelessness and a lack of meaning in life, indicative of existential loneliness rooted in isolation from sources of meaning and purpose. Such statements may reflect the severity of their loneliness but can also indicate (major) depression and/or existential distress related to the end of life and must therefore be interpreted with caution. Loneliness and depression overlap substantially and can mutually reinforce each other, but they are distinct clinical concepts: loneliness is a highly subjective experience of a lack of social connection, whereas depression is a clinical disorder defined by multiple diagnostic criteria. The data in this study are based on narrative reports without formal psychiatric assessment and cannot by themselves determine the prevalence or probability of depression or suicidal ideation in the sample. Nevertheless, the findings have important clinical implications for home care. When patients express a wish to die or persistent meaninglessness, it should prompt assessment with validated screening tools and suicide risk evaluation by qualified healthcare professionals, and rapid referral to appropriate services when indicated. Home care services must therefore have clear procedures for risk assessment, documentation, collaboration with the general practitioner/mental health services, and training in how to handle such conversations in a safe and ethically responsible manner. One study noted that loneliness was one of the main factors associated with older community-dwelling adults’ wish to die, alongside other factors such as depression, social isolation, living alone and low social participation. Subjective loneliness was nevertheless the strongest risk factor for a wish to die. However, this is not evidence that loneliness alone causes a wish to die: the data are cross-sectional, and depression is a very strong co-occurring factor. The study’s authors also note that interventions targeting loneliness and depression may reduce the wish to die [45]. When loneliness becomes so intense that it brings a sense of meaninglessness, often accompanying the approach of death, it may be expressed as “no point in being here anymore”, which may be consistent with the experience of existential loneliness [13]. These results concur with previous research highlighting the link between isolation and poorer mental and physical health in older patients [46,47].

Care that is impersonal and poorly delivered not only reduces opportunities for meaningful social contact (increasing social loneliness) but also undermines recognition, emotional support and a sense of belonging, thereby particularly exacerbating emotional loneliness. Conversely, respectful, empathetic and consistent care encounters can serve both as social contact and as an emotional lifeline: they foster attachment, trust and the experience of being seen, which alleviates emotional loneliness [48]. Communicating on a deeper level might also decrease experiences of existential loneliness [13], and research shows the significance of everyday conversations as a key element in building trust, promoting a sense of security and safeguarding autonomy [49]. Patients have a desire for home care staff to take the time to get to know them, not only to address medical needs but also to listen to their life stories and recognize their personhood [40,41]. However, time constraints remain a significant barrier [50]. Despite the communication challenges, the care received was generally valued and appreciated. Home care was often described as essential to enable participants to continue living at home, although the results also emphasize a strong desire for greater predictability, consistency and deeper connections. Introductions at the door and interactions marked by friendliness and genuine interest were reported to reduce feelings of loneliness. Meeting these needs requires a concerted organizational effort to foster a culture of respectful, compassionate and open communication. The behaviour and communication of home care staff strongly shape patients’ sense of safety; staff should therefore be trained to recognize signs of loneliness and communicate with patients to alleviate feelings of loneliness. However, this recommendation is based on small-scale (*n* = 11), qualitative data, and training should therefore be implemented alongside organizational changes and evaluated for its actual impact on patient-reported loneliness and safety [51].

This study reveals that loneliness was formed by both positive and negative encounters. Loneliness emerged as a central theme, often leading to diminished quality of life and, in some cases, distress. The experience of loneliness is consistent with previous studies that point out that older people who experience loneliness describe it as painful and find that it disconnects them from other people and from society in general [42,46,47]. This reflects social loneliness characterized by weakened social networks and participation. The present study highlights a need for interventions to prevent loneliness. However, a scoping review that shows the importance of developing such interventions also reveals that existing studies vary considerably and use inconsistent outcome measures. Therefore, there might be a need for well-designed, theory-based trials that specify the target group’s needs and practical implementation conditions [52]. Nevertheless, interventions have modest effects, although some interventions can reduce loneliness. However, because the interventions, participants, methods and measurements in these studies vary widely (and because the effects are often small), it is uncertain how strong and long-lasting the effects actually are [53]. On the other hand, interventions such as activity centres for older adults might increase social connectedness and a sense of belonging, which would strengthen self-esteem and reduce loneliness [10]. In Scandinavia, however, such interventions are not generally offered to older adults living alone [43,47]. Under Norwegian legislation, municipal tasks include preventing disease, injury and social problems and promoting health [22], but do not contain any obligation to provide day centres for older people who experience loneliness [47]. Although many participants felt lonely and empty and questioned the value of life, others found comfort in solitude. For these people, being alone provided space for reflection and a sense of autonomy and freedom [7,44,54]. The present study particularly emphasizes the feeling of living in freedom. This is consistent with results that independence is a valued aspect of living alone, and that solitude is frequently regarded as a preferred state that can provide a sense of meaning [44]. Older patients may thus experience living alone both as a challenge [40,41,42] and as a source of personal strength and satisfaction [6,7,44,54,55]. Factors such as personality, coping style, life history and social context all influence whether living alone is experienced as loneliness or as chosen withdrawal and solitude. Home care could conduct comprehensive assessments to gain insight into whether living alone is desirable for individual older people or whether it will lead to loneliness. By conducting these assessments [56], preventative interventions can help reduce feelings of loneliness. This calls upon home care management to facilitate assessments and place greater emphasis on the mental health of patients receiving home care. This approach also aligns with relevant legislation [57,58].

Small practical gestures, such as folding clothes or taking out the rubbish, were deeply appreciated and had a significant impact, particularly for individuals with limited mobility. As age increases, greater attention is paid to practical matters because declining mobility makes everyday tasks more demanding. Beyond easing daily routines, these gestures conveyed attention and respect, making patients feel seen and acknowledged. Connection and attentiveness can reduce loneliness in older people. However, loneliness remains to be investigated in patient-centred outcome studies [59]. Deteriorating physical health can become a major factor leading to loneliness, as illustrated by the narratives in this study, primarily resulting in social loneliness by restricting mobility and social participation. Home care staff often engage in small, compassionate acts despite time pressure, recognizing their emotional and symbolic value [56,60]. The study by Larsson et al. [60] provides valuable insight from home care nurses showing that short, task-oriented encounters may undermine patients’ sense of safety. However, their conclusions are based solely on nurses’ reports from one municipality (*n* = 11), and an absence of patient perspectives limits transferability. Nevertheless, this study shows that the staff were sometimes experienced as distant, rushed, or unwilling to go beyond formal tasks. Some of the staff even asked participants not to mention their extra efforts to others, reflecting a tension between personal commitment and professional boundaries. This study revealed considerable differences between home care staff; some helped a great deal with practical matters beyond their clinical tasks, while others did not. As previous studies have shown, when patients perceive home care staff as disengaged or stressed, they may feel invisible, burdensome and devalued, which negatively affects their experiences [37]. Research shows that for older adults to thrive, they need to feel that they are not “objects of care”. They need and want to act freely and live an authentic life, even while receiving home care. However, the study in question included only a few participants (*n* = 10) from one municipality. Although the study highlights important existential dimensions of home care and points to the need to promote “existential freedom” and authenticity, there is little discussion of recommendations for practice [61].

In the present study, patient needs often differed from what home care typically provides, creating a gap that led to a distance between patients and staff that could increase loneliness. Encounters were frequently rushed, reflecting alienation that exacerbated loneliness, involving a feeling of being overlooked, devalued, and disconnected from others, experiences that align with the criteria for social and emotional loneliness [8,10]. Social loneliness is largely shaped by how society is organized, and emotional loneliness is likewise socially structured, the key point being that both forms of loneliness are produced by social structures, not only by individual circumstances [12]. While it can be debated whether some expectations of home care recipients are unrealistic, many participants experienced profound loneliness that could have negative health consequences if left unaddressed [18]. The present study also found that even when time permitted, staff sometimes prioritized other activities, such as using their phones instead of engaging in conversation, which participants found demeaning, feeling that it confirmed their exclusion from others despite sharing the same room, as described in other research [13]. There was a profound need for security, continuity, clearer routines and relational presence, with these factors being seen as essential for addressing feelings of loneliness. A key finding in the current study was the lack of continuity, with constant changes in the home care staff visiting the participants, which challenged their sense of security and made it difficult to establish trusting relationships. The importance of the need for security is supported by research [54], while the participants in this study felt secure in knowing that home care staff visited them and that they would receive the help they needed. The patients became distressed when they did not know when home care would visit them, especially given the large number of different staff involved. Such unpredictability and lack of continuity erode trust and reduce opportunities for meaningful interaction, often increasing loneliness, withdrawal from social contact, and poorer overall well-being, and can therefore be understood in relation to both social and emotional loneliness [51,62]. While home care often prioritizes physical needs while failing to meet psychosocial and emotional needs, this study shows that regular, socially oriented services are more effective at reducing loneliness. This is consistent with the view in a study that points out that fragmented, task-oriented or irregular home care, by limiting opportunities for meaningful, predictable contact, plausibly undermines trust and may exacerbate social and emotional loneliness [63]. Dependence on care was experienced as being treated like a task to be performed rather than receiving the undivided mental attention participants needed [64]. In the study by Chua et al. [65], loneliness was common among patients receiving home care. It was associated with living alone, reduced physical functioning, difficulty in meeting daily needs, and poor emotional well-being. The authors underscored the need for holistic care that addressed social and emotional dimensions as well as physical needs.

This study demonstrates a clear gap between the services provided and the needs of older home-dwelling patients. Even when care instructions focus on practical and physical needs, this study shows that patients also want to be acknowledged through good relationships with home care staff involving attentive communication, sufficient time and empathy.

### 4.1. Strengths and Limitations

To ensure trustworthiness, this article aimed to provide a comprehensive description of the study and included quotes that accurately reflected the participants’ perspectives and experiences. Dependability was addressed by adhering to the analytical steps outlined by Lindseth and Norberg [24]. The research team actively reflected on their preunderstandings to minimize bias and accurately represent participants’ perspectives, with reflexivity supporting open, exploratory data collection [32]. Reflexivity was regularly addressed throughout the research process, with careful attention paid to researchers’ assumptions and personal biases, aiming to strengthen the study’s trustworthiness. All members of the team were involved in the analysis and played a role in shaping the article. Three of the researchers in the research team are nurses, and one of these has some experience of working in home care. In view of this nursing background, which involved bringing nursing knowledge and conceptual frameworks that could influence how participants’ statements were interpreted, it was important to remain close to the text. To mitigate the risk of bias, the researcher (BH) documented reflexive field notes after each interview. Initial coding and theme development were performed by BH and discussed regularly with the team of supervisors (RM, KH, LSØ). Theme labels and code definitions were revised iteratively until a consensus was reached.

Participants were offered a transcript review (member checking) [32], which increases trustworthiness. One participant returned a transcript with no objections. The sample included patients from eight municipalities in eastern Norway (urban and rural areas). The study had a small sample size of 12 participants but provided thick descriptions that led to an in-depth understanding of older adults’ lived experiences of loneliness. Recruitment by home care managers may have introduced selection bias (e.g., preferentially inviting satisfied patients). However, the findings do not clearly support this concern. Several participants provided critical and negative assessments of the home care service, and many reported dissatisfactions. This suggests that managers generally adhered to the inclusion criteria and did not systematically select only participants likely to deliver positive accounts. Nevertheless, selection bias remains a possible limitation. It is conceivable that managers assumed certain patients were satisfied and therefore invited them, which could have skewed the results towards more positive accounts. Exclusively positive interviews would probably have produced less nuanced results that did not reflect the complexity of patient experiences. Eight municipalities were included, and it cannot be excluded that staff in one or more of these selected participants whom they expected to provide favourable descriptions of home care. Although some responses were positive, the presence of varied and nuanced accounts argues both for the authenticity of participants’ experiences and against a simple, uniform selection bias. Apart from clearly specifying inclusion and exclusion criteria, no additional measures were implemented to reduce potential recruitment bias.

An important limitation of this study is that participants, when referring to home care, did not specify nurses, and home care includes assistants, healthcare workers, and nurses with different roles and training. Grouping them separately may compromise precision, and the results should therefore be interpreted as applying to Norwegian home care services in general, not to nurses specifically. “Patient” has generally been used when referring to home care in general or in other studies, while “participant” has mainly been used to describe those taking part in the study. This is a small exploratory study, and although the participants lived in different municipalities, larger samples and studies are needed to assess the generalizability of the results.

### 4.2. Suggestions for Further Research and Recommendations for Practice and Education

There is a need for further, more comprehensive research on how home-dwelling older adults receiving home care experience daily life, with an emphasis on interventions that reduce loneliness [52]. Such interventions could promote regular social contact, facilitate participation in community activities, and provide tailored support to strengthen social networks. Research focusing on sustainable healthcare services for older people with frailty is warranted to develop care models that are both effective and enduring. Studies suggest that informal, cost-effective support programmes may enhance well-being and therefore merit further exploration [65].

## 5. Conclusions

This study describes complex experiences of loneliness for most participants, often exacerbated by reduced mobility and shrinking social networks. The patients’ core needs are safety, continuity, and to be treated as whole persons. This means having a small number of designated contact persons and predictable visits that foster trust, open communication, and opportunities to express loneliness. Home care should implement routine screening using short, validated tools and provide targeted follow-up to capture loneliness early and offer individually appropriate interventions. Training programmes in empathetic communication and practical support to connect patients with social meeting places or other relevant services can produce tangible improvements in daily life. Municipal healthcare management should prioritize relationship-building, communication skills, sufficient time for conversation, and organizational solutions that enable continuity and flexibility. Focusing on the patient perspective in planning and evaluation will yield better targeted interventions and support dignified ageing. Future implementations should be evaluated using patient-reported experiences to ensure that they truly meet patients’ needs.

## Figures and Tables

**Table 1:** Participant characteristics.

	Urban or Rural	Gender	Age	Number of Years Received Home Care	Number of Years Lived Alone
Interview 1	Urban	Female	83	39	25
Interview 2	Urban	Male	81	3	35
Interview 3	Urban	Male	94	7	3
Interview 4	Urban	Male	90	6	15
Interview 5	Urban	Female	94	2	94—entire adult life
Interview 6	Urban	Male	88	2	20
Interview 7	Rural	Female	75	15	10
Interview 8	Rural	Female	84	7	25
Interview 9	Urban	Female	80	1	4
Interview 10	Rural	Male	85	1	5
Interview 11	Rural	Female	74	2	15
Interview 12	Rural	Female	98	10	98—entire adult life

**Table 2:** Examples of the structural analysis process.

Meaning Units	Condensation	Sub-Themes	Themes	Main Themes
So, the day passes, but the loneliness is overwhelming and painful. That’s how I feel. For me, the loneliness takes up a lot of space.	Expresses a deep sense of loneliness, isolation and accompanying pain. The loneliness is overwhelming and dominates daily life and has become a constant feeling of emptiness.	A feeling of overwhelming loneliness and the burden of loneliness. Loneliness is a persistent feeling.	Loneliness	An overwhelming and painful feeling
I feel like they’re looking down on me. They enter quickly and just do what is needed. I need eye drops put in, and the home care staff must stay in my home for 15 min to observe the effect. During that time, some of them pick up their cell phones and just sit here, busy with their phones. I wish they could talk to me instead	Staff act in a rushed and disengaged manner during visits. The patient felt looked down on and would prefer conversation and attention while they monitor the treatment	Task-oriented care Perceived disrespect and disengagement Unmet need for relational care and attentive monitoring	Lack of relational care	A presence without connection

## Data Availability

The data is not publicly available. The qualitative interview data contains sensitive personal information, such as locations and illnesses, and the consent obtained from participants did not include public access. Further, the research ethics committee required that the data be safeguarded to protect participant confidentiality. For these reasons, raw data is not publicly available.

## Public Involvement Statement

No public involvement in any aspect of this research.

## Guidelines and Standards Statement

This manuscript was drafted against the COREQ (Consolidated Criteria for Reporting Qualitative Research) checklist [66].
